# Rapid Growth of Nanostructured Diamond Film on Silicon and Ti–6Al–4V Alloy Substrates

**DOI:** 10.3390/ma7010365

**Published:** 2014-01-13

**Authors:** Gopi K. Samudrala, Yogesh K. Vohra, Michael J. Walock, Robin Miles

**Affiliations:** 1 Department of Physics, University of Alabama at Birmingham, Birmingham, AL 35294, USA; E-Mails: ykvohra@uab.edu (Y.K.V.); mwalock@uab.edu (M.J.W.); 2 Laser Inertial Fusion Energy, Lawrence Livermore National Laboratory, Livermore, CA 94550, USA; E-Mail: miles7@llnl.gov

**Keywords:** MPCVD, nanostructured diamond, film growth, thin film structure and morphology

## Abstract

Nanostructured diamond (NSD) films were grown on silicon and Ti–6Al–4V alloy substrates by microwave plasma chemical vapor deposition (MPCVD). NSD Growth rates of 5 μm/h on silicon, and 4 μm/h on Ti–6Al–4V were achieved. In a chemistry of H_2_/CH_4_/N_2_, varying ratios of CH_4_/H_2_ and N_2_/CH_4_ were employed in this research and their effect on the resulting diamond films were studied by X-ray photoelectron spectroscopy, Raman spectroscopy, scanning electron microscopy, and atomic force microscopy. As a result of modifying the stock cooling stage of CVD system, we were able to utilize plasma with high power densities in our NSD growth experiments, enabling us to achieve high growth rates. Substrate temperature and N_2_/CH_4_ ratio have been found to be key factors in determining the diamond film quality. NSD films grown as part of this study were shown to contain 85% to 90% sp^3^ bonded carbon.

## 1. Introduction

Depending on the gaseous chemistry and the growth conditions used, diamond grown by chemical vapor deposition (CVD) can be mono [1,2], poly [3] or nano crystalline [4]. The material properties such as thermal conductivity and electrical conductivity differ between the various crystalline forms of diamond. But owing to some of its other excellent qualities such as chemical inertness and hardness, diamond is a material of choice for a diverse range of applications such as heat sinking, polishing agent in industries; and for scientific applications such as building micro/nano-electromechanical systems (M/NEMS) devices, high pressure research, *etc.* Nanocrystalline diamond grown in the presence of inert gas such as argon is shown to contain very low amount of sp^2^ bonded carbon. Researchers have shown that ultra-nanocrystalline diamond (UNCD) films of superior quality with very low (1%–5%) sp^2^ content could be grown by CVD for various applications [5,6]. Diamond films grown in hydrogen rich—carbon lean environments, termed nanocrystalline diamond (NCD) films have higher grain size compared to UNCD diamond films. Because of the extremely carbon lean chemistry employed in such growths, low growth rates of ~1 μm/h were observed. We have in the past demonstrated the possibility of growing nanocrystalline and ultrasmooth nanostructured diamond (USND) films by CVD on Ti–6Al–4V alloy [5,7–10]. In these studies, nitrogen has been added to the traditional H_2_/CH_4_ chemistry. It has been shown in our studies and other works [6,9,11] that addition of nitrogen has an effect on the phase purity of CVD grown diamond. However, the growth rate achieved in our studies and other studies in literature is not very high. The growth of nanodiamond requires continuous renucleation of diamond crystallites and it is a challenge to maintain this condition over long periods of time. This tends to limit the growth rates. We undertook our present study with the aim of growing nanostructured diamond films of high quality at high growth rates. For applications such as heat sinks, wear resistant coatings on biomedical implants, the CVD grown diamond films need to be tens of microns thick. Growing nanodiamond at high growth rates is very important in such cases.

Our immediate goal is to transfer the success of our studies towards target fabrication for Laser Inertial Fusion Energy (LIFE) project. Stringent requirements exist on the target design and specifications [12,13]. A perfectly spherical shell made of low atomic number material which is X-ray transparent is suitable as target for the LIFE project. Nanodiamond meets these demands given its high density and X-ray transparency. These target shells will be filled with deuterium–tritium. A field of X-rays generated by focusing lasers on the hohlraum, which holds the target; will ablate the outer layer of the target and compress the fuel inside to achieve fusion. Four-millimeter nanodiamond spheres with extremely smooth surface with several microns thick walls are required to be used as targets in the LIFE project. It has already been shown that CVD grown diamond could meet such a challenge [14]. The approach taken by these authors is cost prohibitive, because of low growth rate and the subsequent polishing steps involved. We will detail the results of our initial attempts to grow nanodiamond shells later in this paper.

## 2. Experimental Details

The diamond depositions were carried out in two CVD systems. A 2.45 GHz, 6 kW MPCVD (Wavemat, Ann Arbor, MI, USA) chamber has been utilized for experiments on flat silicon and titanium alloy substrates. A copper sleeve has been added to the stock cooling stage on this system. This allowed us to conduct CVD growths at higher microwave powers and chamber pressures. A 30 kW CVD chamber (Vista Engineering, Birmingham, AL, USA) has been used to carry out diamond film growth experiments on silicon spheres. Ti–6Al–4V alloy disks with diameter of 7 mm diameter and approximate thickness of 1 mm were fabricated from stock sheet of Ti–6Al–4V (Robin Materials, Mountain View, CA, USA) and polished to a mirror finish. Silicon substrates of size 3 mm × 3 mm were cut from silicon <001> wafer of 250 μm thickness. Substrates were cleaned by ultrasonic agitation in acetone, methanol, and water followed by seeding. Substrates were seeded by ultrasonic agitation in nanodiamond slurry (International Technology Center, Research Triangle Park, NC, USA), which has an average particle size of 4 nm. Average CVD growth conditions were 1200 W of microwave power, 12 kPa chamber pressure, and a substrate temperature of 850 ± 20 °C. A two color pyrometer has been utilized to determine substrate temperature. It has to be mentioned that in our previous studies, we have typically used a substrate temperature of 750 ± 20 °C for NSD film growth. However, as part of this study we have conducted NSD film growth experiments at substrate temperatures in the range of 700 C to 900 °C. We concluded that 850 ± 20 °C is best for obtaining high growth rate of NSD. Typical growth experiments were carried out for two to three hours. In our initial experiments, we have employed a gaseous chemistry (GC1) of 500 sccm of H_2_, 88 sccm of CH_4_ and 8.8 sccm of N_2_. It is well known that carbon rich chemistries employed in nanodiamond growth by CVD result in higher sp^2^ content in the films [15–17]. With this in mind, we have undertaken a study to find low methane chemistry suitable for our experiments. After multiple experiments in which we have varied the CH_4_/H_2_ and N_2_/CH_4_ ratios and studied the results carefully, we concluded that gaseous chemistries of (GC2) 400 sccm of H_2_, 20 sccm of CH_4_ and 2 sccm of N_2_ and (GC3) 400 sccm of H_2_, 25 sccm of CH_4_ and 5 sccm of N_2_ have been found to be suitable for our experiments.

The surface morphology of the NSD films has been studied by atomic force microscopy and scanning electron microscopy. Raman spectroscopy and X-ray photoelectron spectroscopy have been utilized to examine the sp^2^ content in the diamond films. Raman spectra were collected at room temperature utilizing a 532 nm excitation wavelength laser, A PHI Versaprobe imaging X-ray photoelectron spectrometer (XPS), operating a monochromatic, focused Al Kα X-ray source (*E* = 1486.6 eV) at 25 W with a 100 μm spot size, was used to determine the chemical bonding of the samples. Charge neutralization is provided by a cold cathode electron flood source and low-energy Ar-ions. All measurements were taken at room temperature and at an argon working pressure of 2 × 10^−6^ Pa; the system base pressure is 5 × 10^−8^ Pa. Survey scans were taken at 187.4 eV pass energy, with a 0.8 eV step; high-resolution scans were taken at 23.5 eV pass energy, with a 0.2 eV step. Sweep averaging was used to improve the signal to noise ratio to acceptable limits. The chemical compositions and bonding states of the films were determined using Multipak v9.0. In order to remove surface contamination, the samples underwent 10 min of Ar-ion sputter etching at 500 V accelerating voltage; cratering effects are limited by rastering the ion beam across a 2 × 2 mm^2^ area. This low-energy etch effectively removed surface contamination, without significant ion damage to the NSD films.

## 3. Results and Discussion

**Figure 1.**
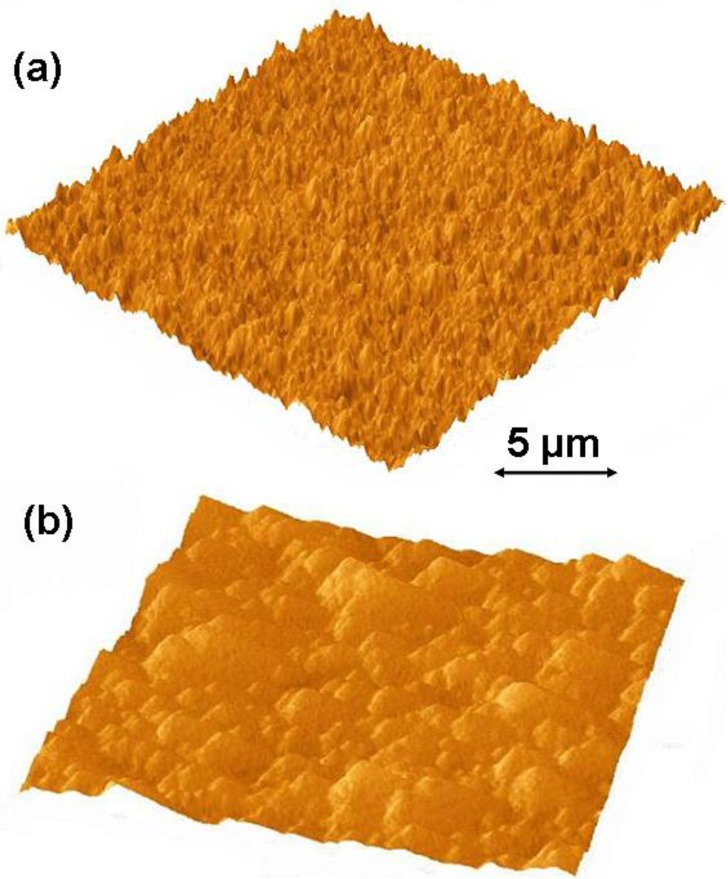
(**a**) Shows the atomic force microscopy image of a 20 μm × 20 μm area of nanostructured diamond (NSD) film grown on Ti–6Al–4V. Root mean square surface roughness = 14 nm; (**b**) Shows the AFM image of a 20 μm x 20 μm area of NSD film grown on silicon. RMS surface roughness = 10 nm. Both NSD films have been grown using GC2 (see text for gaseous chemistry details).

**Figure 2.**
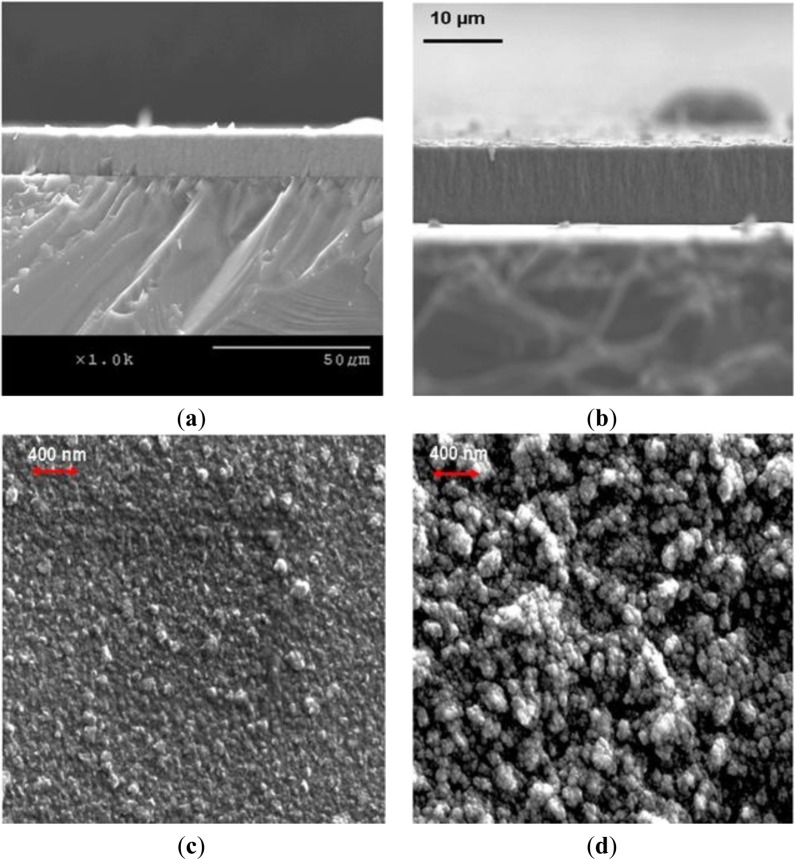
(**a**), (**b**): NSD films grown on silicon substrates using chemical vapor deposition (CVD) chamber pressures of 12 kPa and 9kPa, respectively; (**c**), (**d**): Surface morphologies of NSD films grown using GC1 and GC2 gaseous chemistries, respectively.

Growth rates of 1 to 1.5 μm/h on both silicon and titanium alloy substrates were achieved when experiments were carried out at substrate temperatures of 750 ± 25 °C utilizing the stock cooling stage and gaseous chemistry GC1. The stock cooling stage is the stainless steel cylindrical element with connections to a chiller. Its main purpose is to absorb heat from substrate holder. We noticed that it did not act as a very good heat sink. We could reach our target substrate temperatures utilizing very low microwave power and chamber pressures. Hence, we have added a copper sleeve to the stock cooling stage. As a result, we have noticed dramatic improvement in the heat dissipation capacity of the stage. We could now carry out NSD growth experiments using much higher microwave power and chamber pressures. After installing a copper sleeve and carrying out CVD growth experiments utilizing the plasma with a higher power density, we have managed to achieve a growth rate of 4 μm/h on silicon, and on Ti–6Al–4V substrates. Further experiments to determine substrate temperature’s effect on NSD growth rate were carried out. We concluded that CVD depositions at substrate temperatures of 850 ± 20 °C are beneficial, and at these substrate temperatures we have achieved our highest growth rates of 5 μm/h on silicon. Figure 1 shows the AFM images of NSD films grown as part of this study on (a) Ti–6Al–4V and (b) silicon. Figures 2a,b show the cross-section SEM images of NSD films on silicon that are 10 μm and 7.5 μm thick respectively. Although similar N_2_/CH_4_ ratios have been used in both experiments, the film in Figure 2b has been grown with lower chamber pressure. Figure 2c,d show the surface morphologies of NSD films prepared with gaseous chemistries GC1 and GC2 respectively. All four images shown in Figure 2 are from four different samples. The roughness of the NSD films grown on Ti–6Al–4V alloy has always been found to be higher than the roughness of films grown on silicon, even though identical growth conditions have been employed in both cases. One possible explanation could be that the roughness of the intermediate carbide layer on titanium alloy is greater as compared to the roughness of intermediate carbide layer on silicon, which could be affecting the subsequent growth of NSD. Our previous work of growing NSD was done mostly on Ti–6Al–4V alloy. We wished to continue research starting from a known base. Hence we chose Ti–6Al–4V as substrate material in our initial experiments. However, when we switched the substrate to silicon we noticed that both quality and growth rate of NSD films is much better when using silicon substrates. Given that we could obtain good quality NSD films on silicon and the fact that dissolving silicon is much easier compared to Ti–6Al–4V alloy, we chose to continue our studies on silicon.

Optical emission spectroscopy (OES) has been used to study the plasma in order to investigate why growth rate remained largely unaffected with changes in gaseous chemistry. OES by itself cannot provide complete understanding of the CVD growth phenomenon. It can however, provide useful information on the growth radicals present in the plasma. Apart from increases in CN and C2 species concentrations that could possibly be explained by differences in the amount of gases used in the three chemistries, no other information that could explain the similar growth rates achieved with different gaseous chemistries could be gained from OES. A detailed study of surface reactions taking place on the substrate could provide some insight into this phenomenon. Such a study could also explain the reason for differences in surface roughness of films grown on different substrate under identical conditions. The high growth rate of NSD when using low methane gaseous chemistries could be a result of a combination of the following factors—utilizing high power density plasma, optimum N_2_/CH_4_ ratio in the gas mixture, high substrate temperature allowing for diffusion of growth radicals and promoting continuous renucleation.

**Figure 3.**
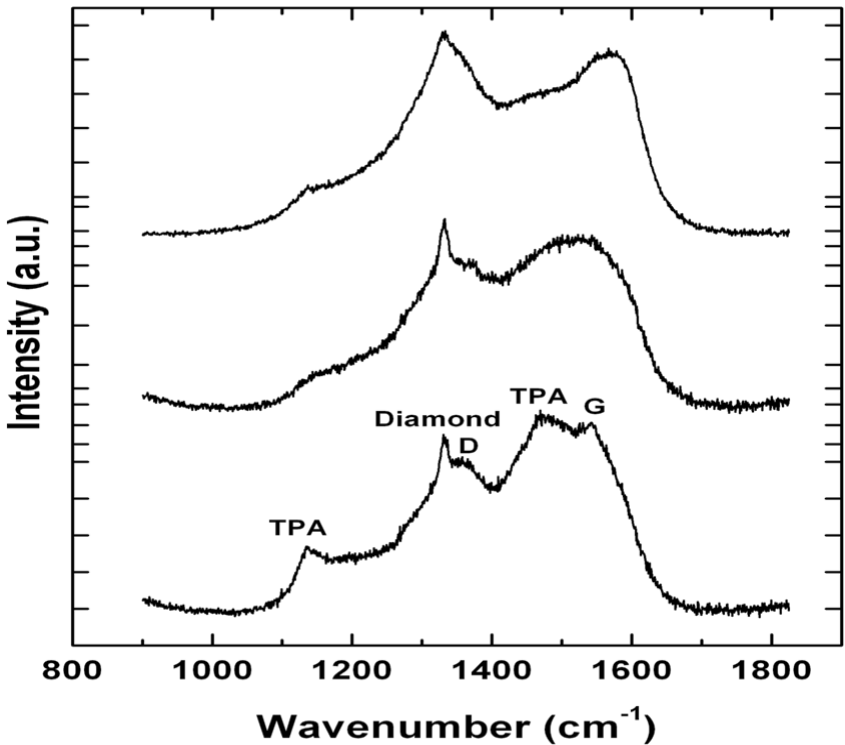
Raman spectra from NSD films grown on silicon using GC1 (bottom), GC2 (middle), and GC3 (top) gaseous chemistries are shown. A low level of amorphous carbon is apparent from the middle, top spectra.

We have observed no significant change in growth rate of the NSD films as a result of utilizing different gaseous chemistries GC2, GC3. This result is very significant given that lower methane usage in gas chemistry is known to produce nanodiamond films with less amorphous carbon. This can be readily seen in our Raman spectroscopy. All spectra shown in Figure 3 have been collected at the same time using identical laser power and collection times. The top two spectra in Figure 3 have been collected from NSD film grown using GC2, GC3 and the bottom spectrum has been collected from NSD film grown using GC1. The bottom spectrum shows the characteristic diamond peak at 1332 cm^−1^, the D, G bands associated with amorphous carbon centered around 1350 cm^−1^ and 1550 cm^−1^, respectively. There are the peaks at 1150 cm^−1^ and 1480 cm^−1^, which are associated with sp^2^ bonded carbon in the form of TPA—transpolyacetylene [18]. All these peaks are still present in the top spectra. However, the TPA peaks have less intensity, which leads us to make a qualitative assessment that it could be due to the presence of low amount of sp^2^ bonded carbon.

**Figure 4.**
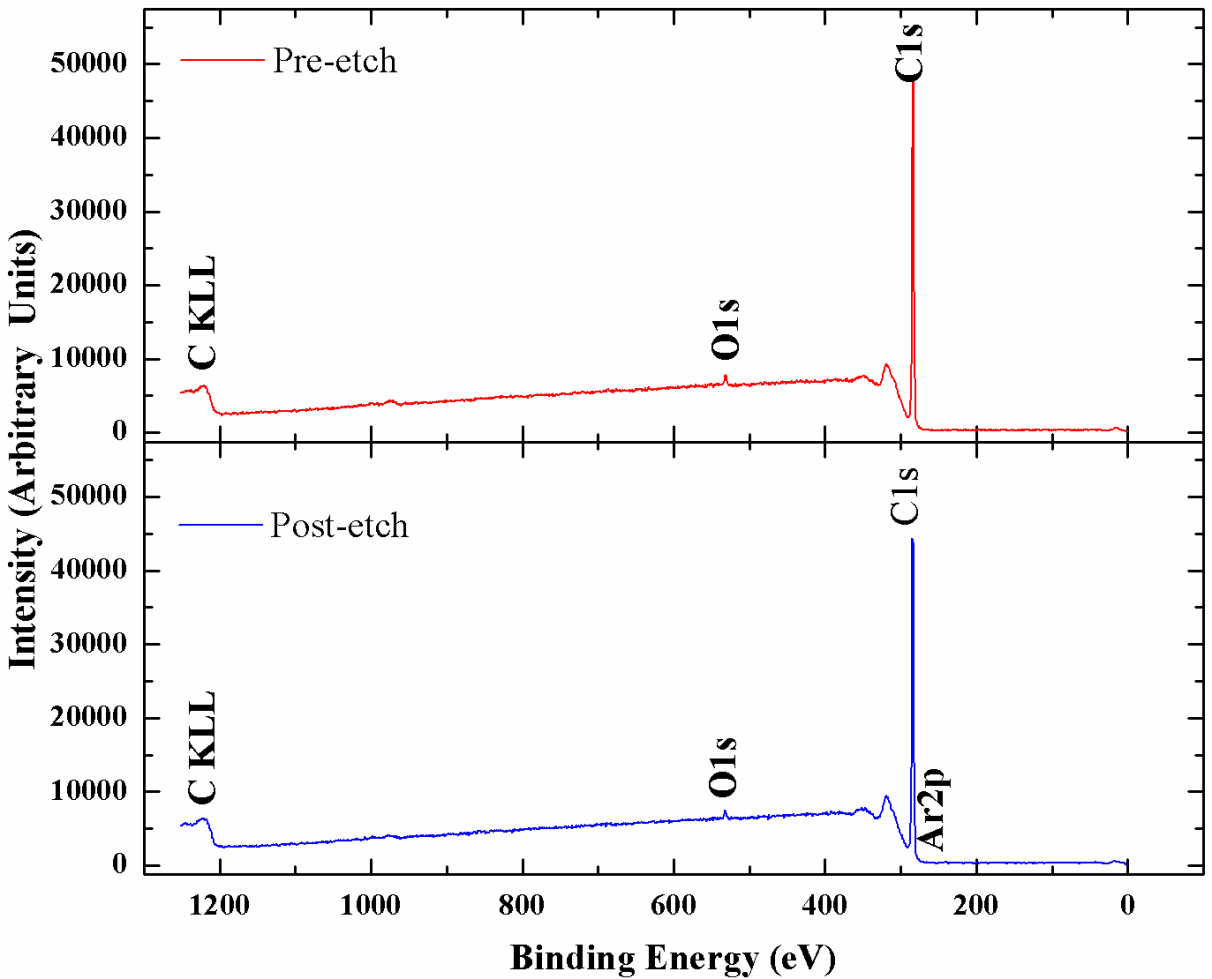
X-ray photoelectron spectrometer (XPS) survey scans of NSD film grown on Ti–6Al–4V utilizing a gaseous chemistry of 500 sccm of H_2_, 88 sccm of CH_4_ and 8.8 sccm of N_2_.

**Figure 5.**
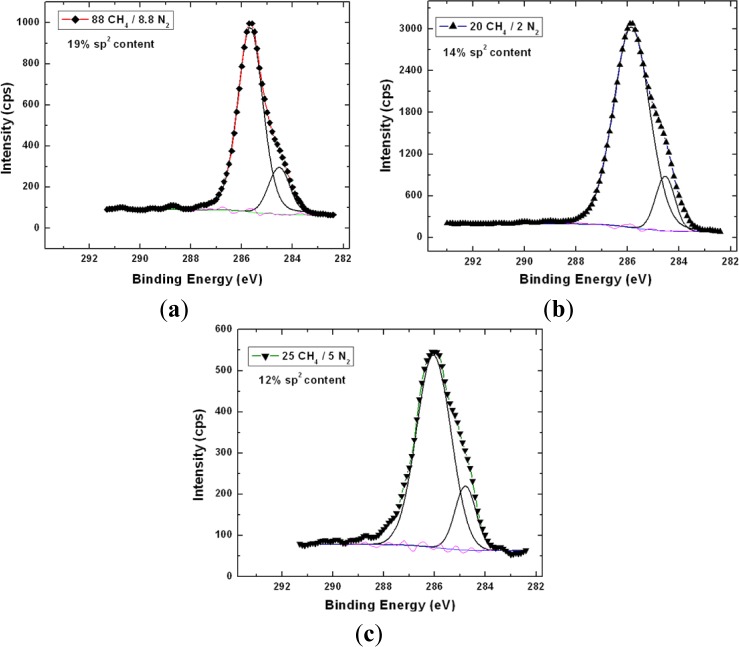
XPS high resolution spectra (C1s) of NSD films grown on silicon with different gaseous chemistries showing different sp^2^ contents. Low methane chemistry resulted in NSD films with less sp^2^ content.

X-ray photoelectron spectroscopy has been utilized to accurately determine the sp^3^ content in the NSD films we have grown. Figure 4 shows the survey scans taken on the NSD film grown on Ti–6Al–4V (pre and post etching). There is no evidence of any major impurities in the film. Especially, no evidence of large amounts of nitrogen or other impurities could be seen confirming that these NSD films are indeed diamond crystals embedded in sp^2^ bonded carbon matrix. However, without conducting a more thorough study we cannot rule out presence of nitrogen in the ppm level. We performed XPS on our samples in order to determine sp^2^ content in our NSD films. When analyzing the data, the residual curve, χ^2^ value, FWHM were all observed carefully to ensure that the fittings are meaningful. As expected, films grown with high methane showed 25% to 30% sp^2^ bonded carbon. Switching to low methane chemistry proved very beneficial, as we have consistently grown films which have only 10% to 14% of sp^2^ bonded carbon as shown in Figure 5. The drop in sp^2^ content is more pronounced in NSD films grown on Ti–6Al–4V compared to the films grown on silicon when utilizing high and low methane chemistries. Efforts are still underway to arrive at ideal growth conditions that would allow us to further reduce the sp^2^ content in the NSD films without sacrificing growth rate. It is a very big challenge given that higher level of methane is associated with high levels of amorphous carbon in CVD grown nanodiamond films. We plan to further investigate the combined effects of N_2_/CH_4_ ratio, substrate temperature to achieve our goal.

**Figure 6.**
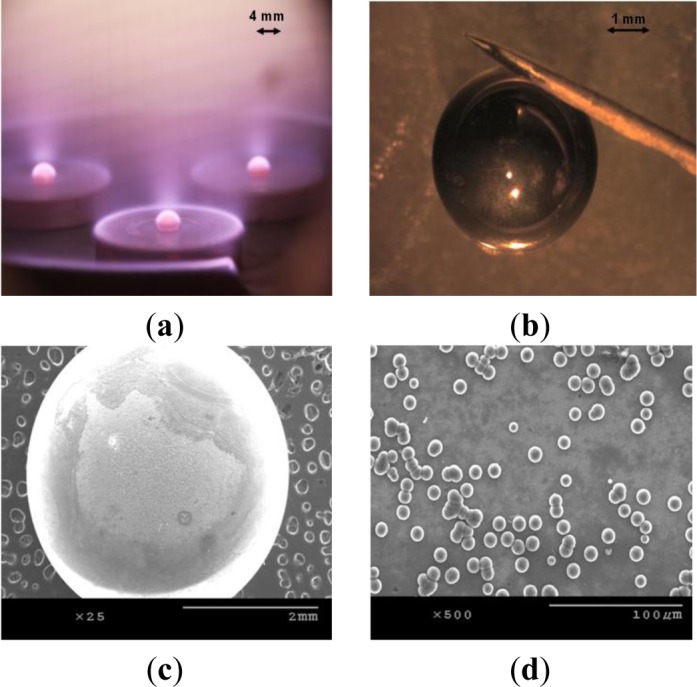
(**a**) Multiple silicon spheres being processed in a 30 kW CVD chamber; (**b**) Nanodiamond coated silicon sphere. Smooth surface is reflecting the needle held close to it; (**c**), (**d**) show areas where non uniform growth of NSD has been observed on silicon spheres.

Efforts are underway to further modify the substrate holder design and substrate cooling mechanism in order to carry out experiments utilizing plasmas with even higher power densities. We have taken our present result and employed it in fabrication of targets for Laser Inertial Fusion Energy (LIFE) project. As mentioned previously, 4 mm nanodiamond spheres with extremely smooth surface with several microns thick walls are required to be used as targets in the LIFE project. We have attempted to grow nanodiamond on silicon spheres obtained from Hauser Optik (Solms, Germany). Figure 6a shows three silicon spheres simultaneously being processed in a 30 kW CVD chamber (Vista Engineering, Birmingham, AL, USA), and Figure 6b shows the resulting nanodiamond coated sphere. We have achieved a growth rate of 2.5 μm/h on silicon spheres. A reason for the drop in growth rate is that we have grown NSD on silicon spheres at 6 kpa chamber pressure, which is considerably less than where we carried out experiments on flat substrates. Our approach in coating the silicon spheres is to coat half of each sphere at a time (as seen in Figure 6a). In a single run when we carried out NSD growth on multiple silicon spheres, not all spheres were uniformly coated. As seen in Figure 6c,d, we sometimes encountered issues with non-uniform growth of NSD on silicon spheres. We are working towards developing a new stage design which would ensure uniform NSD film growth on silicon spheres. We believe our seeding mechanism of ultrasonic agitation in nanodiamond slurry might not be suitable for these spherical samples. In some cases, the spheres were only partially coated with nanostructured diamond. An approach of seeding by a combination of mechanical scratching and ultrasonic agitation [19,20] might prove beneficial in our future experiments.

## 4. Conclusions

In summary, we have developed a stage design and low methane gaseous chemistry that enabled us to grow nanostructured diamond films at high growth rates on silicon and Ti–6Al–4V alloy. Raman spectroscopy, X-ray photoelectron spectroscopy showed that we have grown good quality diamond films. Investigation of low methane chemistries enabled us to consistently deposit diamond films with 85% to 90% sp^3^ content. Initial attempts at growing nanostructured diamond on curved surfaces such as silicon spheres have been partially successful and indicate the direction for future research.
